# Two‐dimensional *in vivo* dose verification using portal imaging and correlation ratios

**DOI:** 10.1120/jacmp.v15i4.4752

**Published:** 2014-07-08

**Authors:** Stefano Peca, Derek W. Brown

**Affiliations:** ^1^ Department of Physics and Astronomy/ University of Calgary Calgary AB; ^2^ Department of Medical Physics Tom Baker Cancer Centre Calgary AB Canada

**Keywords:** EPID dosimetry, treatment verification, *in vivo* dosimetry

## Abstract

The electronic portal imaging device (EPID) has the potential to be used for *in vivo* dosimetry during radiation therapy as an additional dose delivery check. In this study we have extended a method developed by A. Piermattei and colleagues in 2006 that made use of EPID transit images (acquired during treatment) to calculate dose in the isocenter point. The extension allows calculation of two‐dimensional dose maps of the entire radiation field at the depth of isocenter. We quantified the variability of the ratio of EPID signal to dose in the isocenter plane in Solid Water phantoms of various thicknesses and with various field sizes, and designed a field edge dose calculation correction. To validate the method, we designed three realistic conventional radiation therapy treatment plans on a thorax and head anthropomorphic phantom (whole brain, brain primary, lung tumor). Using CT data, EPID transit images, EPID signal‐to‐dose correlation, and our edge correction, we calculated dose in the isocenter plane and compared it with the treatment planning system's prediction. Gamma evaluation (3%, 3 mm) showed good agreement (P_γ<1_ ≥ 96.5%) for all fields of the whole brain and brain primary plans. In the presence of lung, however, our algorithm overestimated dose by 7%–9%. This 2D EPID‐based *in vivo* dosimetry method can be used for posttreatment dose verification, thereby improving the safety and quality of patient treatments. With future work, it may be extended to measure dose in real time and to prevent harmful delivery errors.

PACS numbers: 87.55.km, 87.55.Qr, 87.55.T‐

## I. INTRODUCTION

The goal of radiation therapy (RT) is to deliver the prescribed dose to the target while sparing surrounding tissues. To this aim, the great majority of cancer centers rely on pretreatment quality assurance of the plan calculation by the treatment planning system (TPS) and of the dose distribution in homogeneous phantoms. Nonetheless, there are many arguments in favor of *in vivo* dosimetry (IVD), that is, a method to measure the dose deposited in the patient during treatment, as an auxiliary optimization and safety procedure. IVD can identify errors in dose calculation, data transfer, patient setup, and dose delivery, and may be used as a trigger for adaptive radiotherapy in cases of changing patient anatomy.^1^, ^2^, ^3^ More importantly, most RT errors which have led to serious patient injury or death^4^, ^5^, ^6^ could have been avoided or reduced with IVD.

Currently, the two most common methods of *in vivo* dosimetry available are thermoluminescent dosimeters and diodes; however, both have a number of limitations. The placement of the device on the patient and the readout procedure are time‐consuming, prone to error, and require additional resources. The acquired measurement represents only one point in space and provides only surface dose (or at depth 1−2 cm using buildup). In addition, the presence of any device on the patient's skin may modify the surrounding dose distribution.

Another tool for *in vivo* dosimetry, which has been largely investigated but used clinically only in few select sites,^(1)^ is the electronic portal imaging device, or EPID. The intensity of the transit portal image (acquired through the patient during treatment) can be related to the dose absorbed by the patient. Amorphous‐silicon (a‐Si) EPIDs, in particular, have desirable dosimetric properties including linearity with dose, nondependency with dose rate, and good reproducibility.^7^, ^8^, ^9^, ^10^, ^11^ Additional strengths of the EPID as a dosimeter are: it is readily available, easy to operate, and can produce two‐dimensional dose maps. Finally, the EPID can be run in continuous acquisition or cine mode, and thus has potential to provide dose measurement in real time.^(8)^

Possibly the major contributions to clinical EPID‐based dosimetry come from Netherlands Cancer Institute group, which was able to produce 2D dose maps inside a phantom^(12)^ and translate the method into routine clinical practice.^(1)^ In its first 4.5 years since implementation (2005‐2009), treatment plans of 4337 patients have been verified and 17 serious errors that led to intervention were detected, of which nine would not have been caught by pretreatment verification.^(1)^ Although the clinical results are very good, the method also has some drawbacks. Firstly, it requires extensive commissioning, including ion chamber profile measurements for various field sizes. As well, it does not account for: beam flatness variation with depth, ghosting,^(13)^ and signal dependence on energy spectrum.^(14)^ Regarding the latter, the energy spectrum which reaches the imager is different for each pixel due to differential beam hardening from the flattening filter and from the patient. The ${\text{Gd}}_{2}O_{2}S$ phosphor scintillator of the detector is not water‐equivalent; at lower energies its higher equivalent Z increases the probability of photoelectric events, causing a larger response. Lastly, the method does not account for tissue inhomogeneities, although a variation was later proposed to circumvent this limitation.^(15)^

On another front, a number of groups have modeled the response of the EPID for dosimetric purposes using Monte Carlo techniques.^16^, ^17^ One group, in particular, was able to calculate accurate 2D dose maps inside a phantom by means of sophisticated EPID modeling.^18^, ^19^ Pure simulation and mixed simulation/empirical methods can provide very accurate results, but require highly specific mathematical models for both the accelerator and the EPID. As well, the long calculation times can render them inapplicable to clinical routine (up to 336 hours for a single volumetric modulated arc therapy plan^(20)^).

Another group has investigated IVD using the EPID modified to direct detection with promising results, but presently not applicable with ease to clinical routine as it requires replacement of the phosphor screen with solid water.^(21)^ Kavuma et al.^(22)^ developed a promising method for IVD using EPID images and depth‐dose data. Cine‐mode EPID imaging has found application in the realm of gantry motion verification for dynamic RT techniques^23^, ^24^ and as a pretreatment dose verification tool,^8^, ^25^ but real‐time EPID IVD is not current clinical practice. For further applications of portal imaging IVD the reader is referred to comprehensive review papers available in literature.^2^, ^11^

Although much research has been done in the field of EPID IVD, the methods described above are not easily applicable in the clinic. In regard to commercial applications, the only available option is Dosimetry Check with the exit‐transit dose option (available through Oncology Systems Limited, Shrewsbury, UK, and through Math Resolutions, Columbia, MD). Although its pretreatment verification has been documented,^26^, ^27^ the *in vivo* option lacks peer‐reviewed publications. For these reasons we decided to develop a fast, easy‐to‐implement, and clinically reliable method of two‐dimensional EPID IVD.

The *in vivo* dosimetry method we propose is a two‐dimensional extension of previous work by Piermattei et al.^(28)^ which has successfully implemented transit EPID dosimetry to calculate absorbed dose at the isocenter to within 3% of the value predicted by the TPS. Their method relies on a set of correlation ratios which must be determined in advance, and takes into account the impact of tissue inhomogeneities on the primary component of the radiation beam by use of computed tomography (CT) data. Further work has enabled this group to implement their *in vivo* dose‐at‐isocenter verification in various centers in Italy^(29)^ and to apply it in numerous treatment situations.^30^, ^31^, ^32^

We expanded the work by Piermattei et al. to calculate 2D dose maps in an anthropomorphic phantom during three‐dimensional conformal RT (3D CRT). To this aim, we had to characterize the off‐axis variability of the correlation ratios F, here defined as the ratios of the EPID signals and the TPS doses in equivalent water phantom midplanes.^(28)^ The variability of the F factor is due to multiple effects. Most importantly, moving from the central axis (CAX) to the field edge, the dose decreases more rapidly than the signal due to a major loss of lateral electronic equilibrium in the phantom, thus causing F to increase.^12^, ^33^ A lesser effect is due to the flattening filter which causes beam hardening closer to the CAX. The detector, being nonwater‐equivalent, has a response that is oversensitive to photons of lower energy,^34^, ^35^ producing a higher response for the same dose when farther from the CAX. This effect is actually overcompensated by the flood field correction (acquired with an empty beam), which does not account for the variation in beam attenuation caused by in‐patient hardening.^(12)^ Because these two effects are most likely accelerator‐ and EPID‐dependent, we chose to account for them by an empirical, rather than model‐based, approach. This was done by measuring the values of F in the whole isocenter plane for various thicknesses and field sizes to determine the appropriate correction factors.

The goal of this work is to provide proof of principle for a 2D *in vivo* dose verification method that is simple to implement and to use routinely, and that is sensitive to clinically relevant dose delivery errors.

## II. MATERIALS AND METHODS

**Figure 1:**
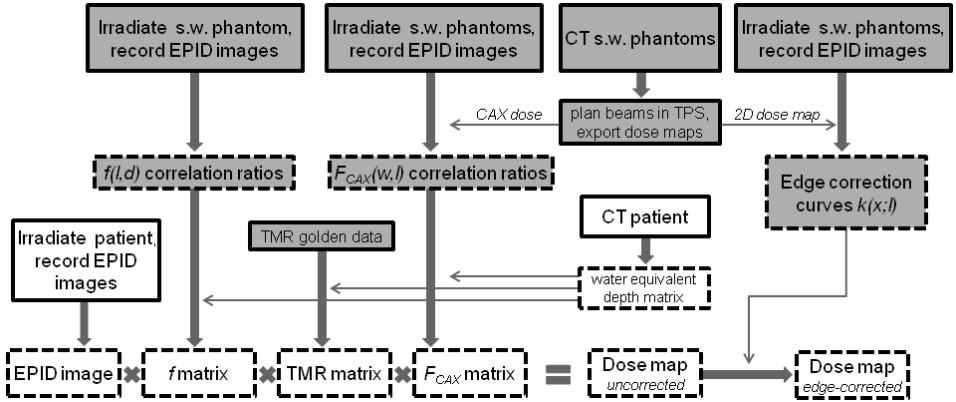
Dose calculation pipeline. Gray=commissioning procedure which has to be run only once; white=dose calculation steps per each patient/field; dashed frame=calculations performed in MATLAB environment; s.w.=solid water; ‘patient’ also refers to anthropomorphic phantom.

A number of measurements and calculations must be performed in order to obtain dose maps from EPID images. Figure 1 provides an overview of the calculation process, separated into commissioning and beam‐specific measurements. A detailed procedure is provided below.

### A. EPID signal to dose correlation ratios

**Figure 2:**
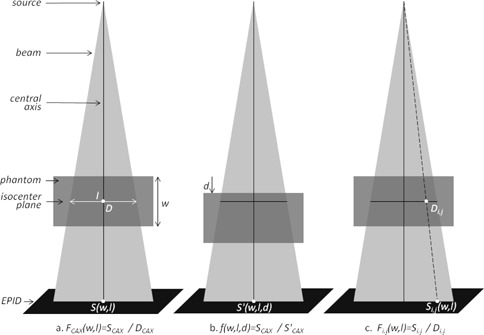
Solid Water phantom setup for measurement of correlation factors $F_{\text{CAX}}$ (a) and *f* ((a) and (b)). Measurements were performed for five thicknesses w, seven field sizes l, and nine displacements d; (c) schematic for off‐axis $F_{i,j}$ measurements.

The correlation ratios $F_{\text{CAX}}$ and the displacement factors *f* were measured by irradiating Solid Water phantoms (Gammex, Middleton, WI) while acquiring transit EPID images, as described by Piermattei et al.^(28)^ In this work, $F_{\text{CAX}}$ values relate central‐axis EPID signal to the dose in the midpoint of the phantom when centered at isocenter (Fig.2(a)), and were determined as the ratio of the signal from the EPID's central 20×20 pixels ($S_{\text{CAX}}$) to the dose at isocenter ($D_{\text{CAX}}$) given by the TPS (Eclipse 8.9; Varian Medical Systems, Palo Alto, CA), for five Solid Water phantom thicknesses (w=6,12,16,20,26 cm) and seven square fields (l=5,7.5,10,12.5,15,17.5,20 cm). Similarly, *f* values account for different scatter photon contributions on the EPID due to displacement of the phantom from isocenter (Fig.2(b)), and were measured as the ratio of the EPID signal with the phantom centered on isocenter to that with the phantom displaced by d, for four field sizes (l=5,10,15,20 cm) and eight displacements (d=−10 to+10 cm in steps of 2.5 cm), on a phantom of thickness 26 cm (because *f* was seen to be independent of w to within 0.3%^(28)^). In this work, the field edge is defined as those points where the portal image has signal equal to half of the sum of $S_{\text{CAX}}$ and of an average pixel value well outside the field.

All beams were delivered on a Varian Clinac 21 EX in service mode (6 MV, 100 MU, 300 MU/min), with the EPID acquiring images in cine mode using the AM (Acquisition Module) Maintenance software (Varian Medical Systems). The EPID was a Varian aSi 1000 with resolution set at 384×512 pixels which produces an image field size of $20.1\times 26.8\;{\text{cm}}^{2}$, and was placed at a source‐to‐detector distance of 150 cm. The EPID frame rate was set to 10 fps, with eight consecutive frames averaged into a single image, resulting in 1.25 images/sec. The detector was read out synchronously to the beam pulse pattern, making the calibration dose rate dependent. All “raw” EPID images are flood field and dark field corrected. For each field, images were exported separately and summed in MATLAB (The MathWorks Inc., Natick, MA).

To investigate the variability of the F factors in 2D we made use of the EPID images described above, determining the ratios

(1) $$F_{i,j}(w,l)=\frac{S_{i,j}(w,l)}{D_{i,j}(w,l)}[a.u/\mathrm{cGy}]$$

for every ray line from the source through the phantom to each detector pixel i,j (Fig.2(c)). Here $S_{i,j}$ are the detector pixel values, and $D_{i,j}$ are point doses inside the phantom in the isocenter plane. The dose calculation was performed with the AAA algorithm with heterogeneity correction. The dose calculation grid was set at 0.25 cm, but to obtain $D_{i,j}$ values, a dose map was exported from the TPS with the same number of points as the detector pixels. Subsequently, we defined unitless $K_{i,j}$ values as $F_{i,j}$ normalized to $F_{\text{CAX}}$:

(2) $$K_{i,j}(w,l)=\frac{F_{i,j}(w,l)}{F_{CAX}(w,l)}$$

**Figure 3:**
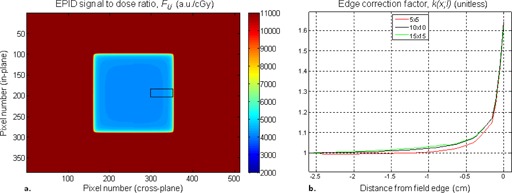
(a) 2D map of the signal‐to‐dose correlation factor $F_{i,j}$. For every pixel i,j of the EPID, $F_{i,j}=S_{i,j}/D_{i,j}$ where S is the signal intensity and D is the dose determined by the TPS in the point where the ray line from the source to the pixel intersects the isocenter plane (Fig. 2(c)). This matrix was calculated for three field sizes (shown: l=10 cm) and for five phantom thicknesses (shown: w=12 cm). The black rectangle contains the 20 cross‐plane subprofiles which were averaged and normalized by $F_{\text{CAX}}(w,l)$ to compute the mean 10±10 edge correction curve. (b) Edge correction curves for three field sizes from the 12 cm thick phantom.

From this 2D matrix, we determined one‐dimensional correction curves k(x;w,l) by averaging 20 cross‐plane subprofiles consisting in the 53 pixels (2.76 cm projected on the isocenter plane) closest to the central‐right field edge, as illustrated in Fig. 3(a). This was done for all values of w and for three values of l (5, 10, 15 cm). Here, *x* refers to the distance from the point *i,j* to the field edge in the cross‐plane direction. We chose this subprofile length because we found that, in all cases, this region contained >99% of the variability of F. Similarly, we chose to average 20 central subprofiles to obtain a mean curve, which well‐described the increasing trend of F in proximity of a single field edge. Mathematically, this step may be written as:

(3) $${〈K_{i,j}(w,l)〉}_{\text{over}\;20\text{ sub‐profiles}}=k(x;w,l)$$

For clarity, the major dependence of F on position in the plane is with the distance to field edge (and only indirectly with distance from central axis). For this reason, it is more appropriate to refer to it as an edge correction factor rather than an off‐axis factor. The purpose of the k(x;w,l) curve is precisely to approximate the behavior of the pixel‐to‐dose ratio at a distance x from a field edge.

### B. 2D dose calculation in Solid Water phantoms

**Figure 4:**
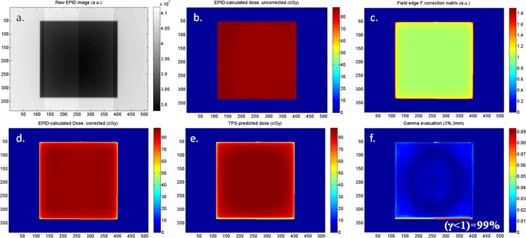
The fundamental steps of our dose map calculation and evaluation: (a) EPID image of 16 cm solid water with $15\times 15\;{\text{cm}}^{2}$ field, continuous acquisition mode; (b) calculated dose using signal‐to‐dose correlation factor from the central axis $F_{\text{CAX}}$ (as previously described^(28)^); (c) field edge correction to $F_{\text{CAX}}$ obtained using the 15×15 curve from Fig. 3(b) and a binary mask of the EPID image; (d) edge‐corrected dose map obtained by pixel‐by‐pixel division of (b) by (c); (e) dose map predicted by the TPS; (f) gamma evaluation (3%, 3 mm) of the dose maps. Passing values (γ<1 for >95% of in‐field pixels) were obtained for all solid water thicknesses and field sizes. Dose maps are masked to field size.

We obtained 2D dose maps in phantoms (Fig. 4) using the $F_{\text{CAX}}$ correlation ratios (e.g., Fig. 4(b)) and then corrected these maps by multiplying by k(x;w,l) and k(y;w,l), where x and y are the distances from the point i,j to the closest field edges in the cross‐plane and in‐plane directions (e.g., Fig.4(c)). Here we are approximating that for any given point of the isocenter plane i,j the edge proximity effect is due to two field edges only (i.e., the closest edges in the cross‐plane and in‐plane directions). Based on our finding that >99% of the off‐axis variability of F is contained in the 2.76 cm from a single field edge, this approximation will hold to <1% for square fields of size greater than about 5.5 cm.

A simple example serves to illustrate this step. Suppose one wishes to know the correct F factor for a point in the top‐right corner of a square field of side 1 incident on a homogenous phantom of thickness w. Said point is x cm from the right edge (cross‐plane direction) and y cm away from the top edge (in‐plane direction). The corrected factor $F_{i,j}$ is then the product of $F_{\text{CAX}}$ by k(x;w,l) and k(y;w,l), or

(4) $$\frac{F_{i,j}(w,l)}{F_{CAX}(w,l)}=k(x;w,l)\cdot k(y;w,l)$$

We then tested the efficacy of the various correction curves k(x;w,l) by gamma evaluation (dose difference: 3%, distance‐to‐agreement: 3 mm) between the EPID‐calculated and TPS‐predicted dose maps on the Solid Water phantoms. Empirically, we found that for the same field size, using the correction curve obtained from solid water of thickness w=12 cm gave good results for all other thicknesses (see Results section below). For any given field l, then, k(x;w,l) can be approximated as k(x;12cm,l) which, for simplicity, we will write as k(x;l). The same consideration applies to k calculated in the in‐plane direction, y. Consequently, for any point i,j we can write the following approximation:

(5) $$\frac{F_{i,j}(w,l)}{F_{CAX}(w,l)}\approx k(x;l)\cdot k(y;l)$$

where the *k* curve is derived from the w=12 cm phantom measurements.

In conclusion, in the case of homogeneous phantoms centered at isocenter, dose in the isocenter plane can be calculated using the relation

(6) $$D_{i,j}^{calc}(w,l)=\frac{S_{i,j}}{F_{i,j}(w,l)}\approx \frac{S_{i,j}}{F_{CAX}(w,l)\cdot k(x;l)\cdot k(y;l)}$$

The variability of the *f* displacement factors in the isocenter plane was also investigated.

### C. 2D dose calculation in anthropomorphic phantom

The variability of the *f* displacement factors in the isocenter plane was also investigated.

Three realistic 3D CRT treatment plans were designed on a thorax and head anthropomorphic phantom (The Phantom Laboratory, Salem, NY): 1) whole brain (WB) irradiation with gantry angles (GA) 90° and 270°; 2) brain primary (BP) with GA 40°, 90°, 140°; and 3) lung tumor (LT) with GA 0°, 40°, 180°. Beams were shaped with a multileaf collimator (Varian Millennium 120 leaf MLC) and by setting the most appropriate collimator angle. Couch angle was 0° for all beams.

The anthropomorphic phantom was imaged by means of a clinical CT scanner (Varian Big Bore) with standard imaging parameters (2 mm contiguous slices, 512×512 pixels, 1.1 pixels/mm). CT data were fed into an in‐house algorithm developed in MATLAB environment, which calculates the water‐equivalent depths (i.e., radiological depths) along every ray line from the source to the isocenter plane and from the isocenter plane to the corresponding EPID pixel. The sum of these two depths is equal to the phantom thickness along the ray line, $w_{i,j}$, and their difference is $d_{i,j}$. With knowledge of these values for every ray line, one may calculate dose in every point of the isocenter plane by extending the central axis calculation proposed by Piermattei et al.^(28)^ to the whole plane at isocenter depth, as follows:

(7) $$D_{i,j}^{calc}(w_{i,j},l)\approx \frac{TMR(\frac{w_{i,j}}{2}-d_{i,j})}{TMR(\frac{w_{i,j}}{2})}\cdot \frac{f(d_{i,j},l)}{F_{i,j}(w_{i,j},l)}\cdot S_{i,j}$$

where *l* is the equivalent square field size for the conformal beam.

The use of tissue maximum ratio (TMR) values in the above equation requires clarification. The ratio of TMRs was previously introduced to compensate for the different water‐equivalent depths upstream and downstream with respect to the isocenter plane.^(28)^ In other words, along the CAX, the TMR ratio shifts the point of dose calculation from the half‐depth in the phantom (w/2) to the level of the isocenter plane (w/2−d). It can be easily verified that the ratio of TMRs calculated in such two points is equal to the ratio of dose values in the same two points, and the inverse square law dependence cancels out.^(28)^ In writing Eq. (7) we are now approximating the dose off‐axis by using the TMR ratio corresponding, not to the phantom's half depth (w/2), but the phantom's half‐depth along the ray line (wi,j/2). We are therefore shifting the point of calculation from half‐depth in the phantom along the ray line (wi,j/2) to the depth of the isocenter plane ($\text{wi},j/2-d_{i,j}$), for the purpose of reconstructing a 2D dose map at isocenter depth. The actual TMR values are those calculated along the CAX and provided by Varian. Using our clinic's off‐axis factors (OAR) for 6X beams, we verified that the TMR values would not change by more than about 1%−2% off‐axis, thus supporting the approximation.

Making use of Eq. (5), we can rewrite this relation as:

(8) $$D_{i,j}^{calc}(w_{i,j},l)\approx \frac{TMR(\frac{w_{i,j}}{2}-d_{i,j})}{TMR(\frac{w_{i,j}}{2})}\cdot \frac{f(d_{i,j},l)}{F_{CAX}(w_{i,j},l)\cdot k(x;l)\cdot k(y;l)}\cdot S_{i,j}$$

Note that the equation above is written for the general case in which the radiological thickness is specific for each ray line (i.e., $w_{i,j}$ and $d_{i,j}$ are be different for each pixel). Agreement between calculated dose maps using Eq. (8) and TPS maps for the anthropomorphic phantom was tested with 3%, 3 mm gamma evaluation.

## III. RESULTS & DISCUSSION

### A. Correlation ratios and dose calculation in Solid Water phantoms

The $F_{\text{CAX}}$ and *f* curves as functions of w and l resemble those reported by Piermattei et al.^(28)^, with the exception that values of *f* were contained within 0.6%, a much smaller variability than the 5% previously reported (data not shown). This is most likely attributable to apparatus scattering differences. The *f* factors for the whole field were always well within 1% of the CAX value; for all practical purposes, from here onwards we approximated *f* to have no off‐axis dependency. $F_{\text{CAX}}$ and *f* curves were obtained by cubic interpolation of the data points, as it provided the best agreement to measurements. It is important to emphasize that, in general, every setup (the specific accelerator, EPID, bunker, energy) will have its characteristic correlation ratios, and values obtained in one situation may not be easily applied to another. The specific dose penumbra, in particular, will greatly impact the edge correction curves.

Values of $F_{i,j}$ increased as expected in proximity of field edges (Fig. 3(b)). This off‐axis trend was similar for all water‐equivalent depths and square field sizes. When applying the edge corrections k(x;w,l), we found the best overall agreement (γ<1 for >95% of points inside field) for all thicknesses (irradiated by the same field size) by using the correction curves derived from the 12 cm phantoms. In other words, for our setup, the field edge behavior of $F_{i,j}$ displayed in 12 cm phantoms well‐approximated that of both thinner and thicker phantoms, supporting the use of Eq. (8) to calculate dose in the anthropomorphic phantom. On the other hand, the field size dependence could not be neglected, as there was no single field size which produced good overall agreement for all other field sizes. Rather, we found that using the curves derived from the closest field sizes gave good agreement. The three curves displayed in Fig. 3(b) were then all that we needed to correct the edges of the dose maps from all water‐equivalent depths and square field sizes, including the anthropomorphic phantom calculations.

As an example, the dose calculation and correction steps for the $15\times 15\;{\text{cm}}^{2}$ field of the 16 cm thick phantom are shown in Fig. 4.

### B. Dose calculation in anthropomorphic phantom

Our algorithm calculated dose for the nine beams we delivered to the anthropomorphic phantom. For the WB and BP plans, all calculated dose maps passed gamma analysis (98.3% and 99.3% for WB, 99.1%, 96.5%, 97.2% for BP). For the LT beams, on the other hand, our calculation consistently overestimated dose by about 7%−9%.

The dose overestimation in lung is likely due to two major effects: tissue inhomogeneity and field shape. Regarding the former, although our model takes into account scatter differences due to displacement of the object along the beam direction, it does not consider scatter differences due to tissue inhomogeneities. Specifically, much of the lung tumor region suffers loss of electronic equilibrium due to the lower density tissue surrounding it, resulting in an effectively lower dose, as compared to our calculation. Additional correction factors could be introduced to account for this, with the drawback that it would make the calculation process more cumbersome to implement in the clinic. It should be pointed out also that the IVD in clinical use at the Netherlands Cancer Institute was not applicable in lung until a method to circumvent the obstacle of tissue inhomogeneity was determined.^(15)^

A further variable that may account for dose overestimation in lung is the irregular shape of the lung fields. Our edge correction method is built to account for proximity of field edges in the cross‐plane and in‐plane directions. In highly irregular fields, on the other hand, many points may be in proximity (<2 cm) of field edges in a diagonal direction. These edges would likely not be well‐accounted for, and thus an incorrect signal‐to‐dose ratio $F_{i,j}$ of these points may lead to dose overestimation.

**Figure 5:**
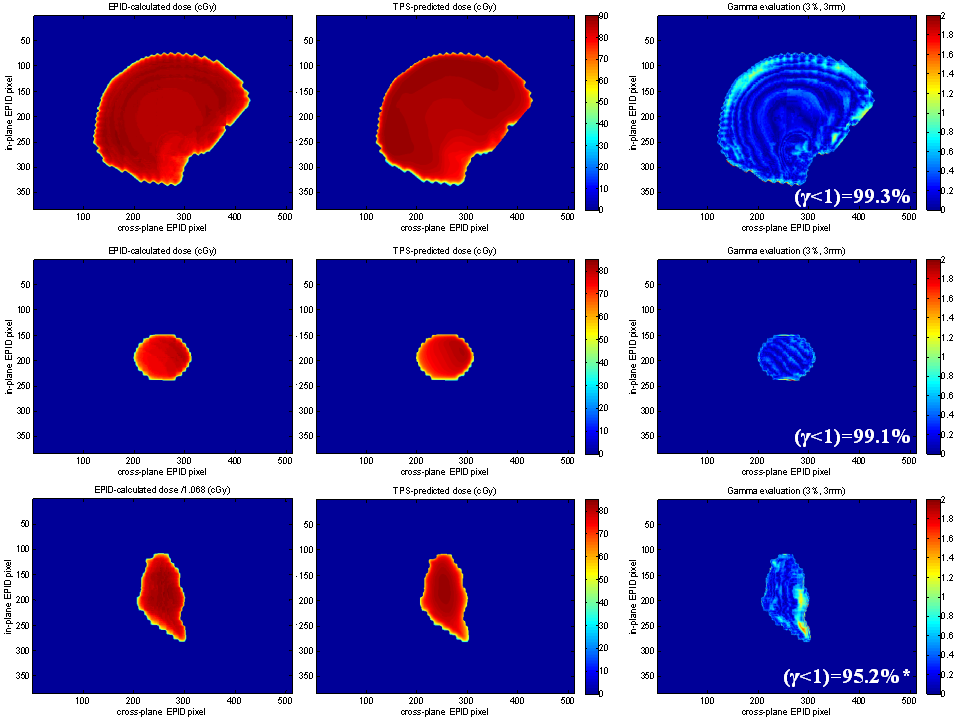
Validation of our dose calculation algorithm on the anthropomorphic phantom. Top to bottom: whole brain with gantry 270°, brain primary with gantry 40°, lung tumor with gantry 0°. Left‐to‐right: EPID‐calculated dose, TPS‐predicted dose, and their gamma evaluation (3%, 3 mm). Edge correction from the curves of Fig. 3(b). Maps are masked to field size. *Note: to provide a meaningful analysis for the lung tumor, the calculated dose has been scaled ad hoc by −6.8% because our calculation overestimated dose in this site.

Figure 5 reports dose calculation results from one field of each of the treatment plans. The left column displays dose maps calculated from EPID images recorded during beam delivery, while the central column reports dose maps exported from the TPS using the same settings as in the clinic.

### C. Limitations

A number of factors may be identified that limit the accuracy of this dose calculation method. Firstly, the edge correction curve has a nonnegligible dependency on thickness and field size and shape. Our approximation of using just three curves to correct maps from all water‐equivalent thicknesses and field sizes will lose some accuracy in very thin or thick regions of tissue, or for more complex field shapes. As well, the approximation of using TMRs (by definition measured along the CAX) to obtain dose at the isocenter depth in points off‐axis may lose validity in presence of high inhomogeneities. These considerations, along with the poor results in lung, suggest that our dose calculation may not be accurate in tumor sites that have high inhomogeneities or require complex field shapes.

It should be pointed out that, in the present work, we did not verify the TPS calculation, but rather validated our dose calculation against it. The TPS dose calculation (including the inhomogeneity correction and CT density table) should be verified independently. In the future, we plan to also use Monte Carlo simulations to produce reference dose maps against which we may compare our calculations. As well, there is uncertainty associated with the Varian TMR data.

It must be noted that all our beams delivered 100 MUs, and it is documented that the dose‐response relationship of EPID tends to lose linearity at low (≤30) MUs.^(36)^ It remains to be verified whether correlation functions obtained at 100 MUs are applicable in the whole clinical range of dose. Regarding our cine imaging modality, we are aware of a source of loss of linearity, for which we did not correct. At the end of each acquisition, there are ‘leftover’ frames which, being fewer than the set frames/image rate, are discarded. This loss of signal becomes more relevant at lower MUs and may become particularly important as we extend the method to intensity‐modulated (IM) RT in which subfields may receive small numbers of MUs. A simple strategy to circumvent this signal loss would be to set the averaging rate to one frame/image, with the drawback that it would increase multifold the number of images with which to work.

Lastly, some unavoidable hardware limitations of any type of EPID‐based IVD should also be stated. Not all beam geometries will allow use of the EPID during treatment, as it may be in collision with the couch, and some very large fields may irradiate the electronics of the detector. In addition, backscatter from the EPID arm can impact pixel signal by up to 6% in the periphery of the detector array^(37)^ and bias results. Finally, increasing many‐fold the use of the EPID may shorten the detector's life span.

## IV. CONCLUSIONS

We have shown that transit EPID dosimetry based on correlation factors (as defined by Piermattei et al.^(28)^) can be adapted to two dimensions and used to estimate dose in the whole isocenter plane for 3D CRT treatment fields.

The main strengths of the method are ease of implementation and speed. Commissioning requires 59 solid water irradiations with EPID transit image acquisitions for a total beam‐on time of just below 20 min (excluding time required for setup adjustments). No ion chamber measurements are needed. Once implemented, the beam‐specific dose calculation is performed in a few seconds, which, combined with the cine imaging, is promising for future real‐time dose verification applications.

This IVD method has potential to be useful in clinical settings, especially when treating regions not in close proximity to large tissue inhomogeneities. The accuracy of the dose calculation is comparable to that used in the clinic for at least some tumor sites. We propose this IVD method not as a substitute to pretreatment QA, but as an adjuvant dose verification, to track dose delivery and to catch serious errors which may be harmful or fatal for patients.

With further work, this method may also be used to evaluate dose distribution variations throughout treatment fractions due to interfractional variability (weight loss, swelling, positioning) and help guide adaptive radiotherapy. We are currently working to improve accuracy and to extend it to real‐time applications and to IMRT. All the MATLAB code is freely available to anyone who will request it by contacting the authors.

## ACKNOWLEDGMENTS

The authors are greatly thankful to Dr. Sarah Quirk and Dal Granville for their contribution in idea generation and data acquisition. As well, we acknowledge Kurt Knibutat for help in image acquisition and Darren Graham for beam planning.
